# A long trip toward REMSs (Residenze per l’esecuzione delle misure di sicurezza): critical issues and perspectives in admitting patients to the Italian forensic psychiatric system

**DOI:** 10.1192/j.eurpsy.2023.1137

**Published:** 2023-07-19

**Authors:** G. Listanti, A. Vaia, L. Baldassarri Hoger Von Hogersthal, C. Romano, D. Gioia, M. Bustini

**Affiliations:** 1 REMS - Dipartimento di Salute Mentale e Dipendenze Patologiche, ASL Rieti, Rieti; 2 Azienda Ospedaliera Sant’Andrea, Roma; 3 Dipartimento di Salute Mentale e Dipendenze Patologiche, ASL Rieti, Rieti, Italy

## Abstract

**Introduction:**

REMSs (Residenze per l’Esecuzione delle Misure di Sicurezza, which roughly translates into Facilities for the execution of security measures) are psychiatric residential facilities introduced in Italy following the discontinuation of Forensic Psychiatric Hospitals, a process started between 2012 and 2014 and concluded in 2017. REMSs are forensic psychiatric communities, focusing on treatment and rehabilitation, managed exclusively by the italian NHS. They host psychiatric patients, who have committed crimes, but are not sended into the ordinary jail circuits because judged as mentally impaired and socially dangerous, with the aim that of stabilizing and treating psychiatric symptoms and that of their gradual social re-insertment. After the closure of psychiatric hospitals in 1978, the overcoming of forensic psychiatric hospitals is the big new thing happening in Italy when it comes to mental health. The introduction of REMSs has spotlighted how much is needed a care program used as a prevention tool, putting the mentally impaired with a social danger profile back into their dignity as human beings. Notwithstanding, after 10 years from its greenlight, some remarkable issues about REMSs system are at hand, starting from long waiting lists, which triggers the double risk of illegal jail detention of the mentally impaired; or leaving free without containment socially dangerous subjects.

**Objectives:**

Purpose of the present study is to offer an overview of the italian REMSs system, focusing on its critical issues such as waiting lists to be admitted and treading prospects for improvement.

**Methods:**

Our work involves a research review on litterature on REMSs, forensic psychiatric services and admitting procedures.

**Results:**

There are 33 REMSs in Italy. As of 31st December 2021, 573 in-patients are hosted in REMSs. The most frequent diagnosis is schizophrenia (33%), followed by personality disorders (32%) and substance abuse (21.4%). 80% of the crimes committed involve violence towards human beings. As of 25th March 2022, the REMSs waiting lists include 605 individuals, 42 of whom were already imprisoned and 561 released. The average waiting time for admittance is about 10 months. Positioning on the waiting list follows the exclusive chronological criterion (date of sentence) and is not related to any clinical risk criteria whatsoever. It is estimated that one third of waiting patients remain without adequate care.

**Conclusions:**

Rethinking the admittance criteria to REMSs is crucial. The use of alternative safety measures, the improvement of community mental health services and a real integration between both legal and heatlh systems in terms of management of the offending psychiatric patient are among ways suggested to avoid breaking the dream of deinstitutionalisation.

**Disclosure of Interest:**

None Declared

